# Rational Defect Engineering via Calcium Doping for High-Efficiency Monolayer MoS_2_ Emission

**DOI:** 10.3390/molecules31173073

**Published:** 2026-09-01

**Authors:** Ying Chen, Guoliang Yu, Yihua Hu, Xin Yang, Youlong Chen, Jingwen Zou, Haoqi Luo, Fangjie Li, Yushuang Zhang, Qing Ye

**Affiliations:** 1 State Key Laboratory of Pulsed Power Laser Technology, Key Laboratory of Electronic Restriction of Anhui Province, Advanced Laser Technology Laboratory of Anhui Province, National University of Defense Technology, Hefei 230037, China; chen_ying@hnu.edu.cn (Y.C.); chenyoulong2@126.com (Y.C.); zoujingwen0103@126.com (J.Z.); luohaoqi@mail.ustc.edu.cn (H.L.); lifangjie25@nudt.edu.cn (F.L.); 2 School of Physics and Electronics, Hunan Normal University, Changsha 410081, China; guoliangyu@hunu.edu.cn; 3 State Key Laboratory of Opto-Electronic Information Acquisition and Protection Technology, Information Materials and Intelligent Sensing Laboratory of Anhui Province, Institutes of Physical Science and Information Technology, School of Physics, Anhui University, Hefei 230601, China; yangxin@ahu.edu.cn

**Keywords:** sulfur-based 2D materials, defect engineering, substitutional doping, giant emission enhancement

## Abstract

Two-dimensional transition metal dichalcogenides (TMDCs) hold great promise for next-generation optoelectronics. However, the low photoluminescence (PL) quantum yield due to inevitable defects during material preparation severely restricts their practical application. Here, we report a rational defect-engineering strategy based on first-principles calculations and realize it experimentally on MoS_2_ monolayers by doping with calcium atoms. First-principles calculations reveal that proper doping can introduce complementary defect levels to effectively tailor carrier dynamics. Guided by this theoretical design, we synthesized calcium-doped MoS_2_ monolayers via one-step chemical vapor deposition. The as-grown doped MoS_2_ flakes reach sub-millimeter scale (~568 μm). Compared with undoped samples, the Ca-doped MoS_2_ exhibits two orders of magnitude PL enhancement, significantly prolonged carrier lifetime, and efficient conversion from negative trions to neutral excitons. This strategy is also applicable to other alkaline earth dopants, providing a generalizable route for defect engineering in two-dimensional semiconductors.

## 1. Introduction

Two-dimensional (2D) transition metal dichalcogenides (TMDCs) have emerged as exceptionally promising candidates for post-Moore electronic and optoelectronic applications, owing to their atomic-scale thickness, tunable bandgaps, strong light–matter interactions, and freedom from dangling bonds [1,2,3,4]. Among the TMDC family, monolayer molybdenum disulfide (MoS_2_) has attracted particular attention due to its direct bandgap (~1.8 eV) at the monolayer limit, making it highly attractive for applications in photodetectors, light-emitting diodes, lasers, and valleytronic devices [5,6,7,8]. However, the practical deployment of monolayer MoS_2_ in high-performance optoelectronic systems faces a formidable obstacle: the intrinsically low photoluminescence quantum yield (PLQY) arising from the high density of native defects inevitably introduced during material synthesis [9,10,11,12,13]. Chemical vapor deposition (CVD), the most widely adopted method for scalable production of monolayer MoS_2_, typically yields materials with substantial sulfur vacancy densities on the order of 10^12^–10^13^ cm^−2^ [14,15,16]. These sulfur vacancies act as deep donor levels below the conduction band minimum, serving as efficient nonradiative recombination centers that severely quench the PL emission [17,18,19]. Furthermore, these defect states provide excess electrons that facilitate the formation of negative trions (X^−^) at the expense of neutral excitons (X^0^), further diminishing the radiative recombination efficiency [20,21,22]. The resulting PLQY of as-grown CVD MoS_2_ is often merely ~0.1–1%, far below the requirements for practical optoelectronic applications [9,23].

To overcome this challenge, numerous defect engineering strategies have been explored, including chemical passivation with superacids [23,24], electrostatic gating [17,25], strain engineering [26], and heterostructure construction [27]. While these approaches have achieved varying degrees of PL enhancement, they often suffer from intrinsic limitations in terms of durability, processability, environmental stability, or scalability [10,11]. Substitutional doping via CVD has emerged as a particularly attractive alternative, offering the potential for simultaneous modulation of both optical and electronic properties in a single-step, scalable process [19,28,29]. However, most prior doping studies on MoS_2_ have focused primarily on carrier polarity modulation [30,31,32], while the PL emission after doping often deteriorates due to the introduction of excess carriers and scattering centers [33,34]. Achieving substantial PL enhancement through substitutional doping—rather than quenching—remains a significant challenge that requires a deeper understanding of defect-level engineering at the atomic scale. Recent studies have demonstrated that doping with group IIIA elements can effectively repair sulfur vacancies, leading to significant PL enhancement and carrier polarity modulation in WS_2_ and MoS_2_ monolayers [20]. These findings validate the feasibility of defect-energy-level engineering and suggest that judicious selection of dopants with suitable electronic configurations could synergistically manipulate defect-induced energy levels to achieve both enhanced emission and controlled carrier dynamics. However, exploration of alkaline earth metals—with their distinctive valence electron configurations—remains largely uncharted in the context of TMDC doping. Among them, calcium (Ca) is particularly attractive: its atomic radius and bonding characteristics are compatible with the MoS_2_ lattice, while its lower valence electron count relative to Mo suggests p-type doping behavior that could effectively neutralize the native n-type carriers arising from sulfur vacancies.

In this work, we report a systematic study of calcium-doped monolayer MoS_2_ (Ca:MoS_2_) synthesized via a facile one-step CVD method, achieving a giant PL enhancement of up to two orders of magnitude. Through comprehensive optical spectroscopy, we demonstrate that Ca doping effectively converts negative trions to neutral excitons by introducing complementary acceptor levels that neutralize the excess electrons from sulfur vacancies. First-principles DFT calculations corroborate the experimental findings, revealing that the synergistic interplay between Ca-induced acceptor levels and native donor levels is the key to the remarkable PL enhancement. The Ca:MoS_2_ samples exhibit a notable blue shift in PL peak positions, reduced full width at half maximum (FWHM), and significantly prolonged carrier lifetimes, all of which are consistent with improved radiative quality and suppressed nonradiative recombination. Our strategy is also found to be extendable to other alkaline earth dopants (such as Mg), suggesting a generalizable defect-energy-level engineering approach for high-performance 2D semiconductors. This work not only provides a practical pathway for dramatically improving the optical quality of monolayer MoS_2_ but also establishes a fundamental framework for designing defect-engineered 2D materials with tailored optoelectronic functionalities for future integrated photonic and optoelectronic systems.

## 2. Results and Discussion

**Figure 1:**
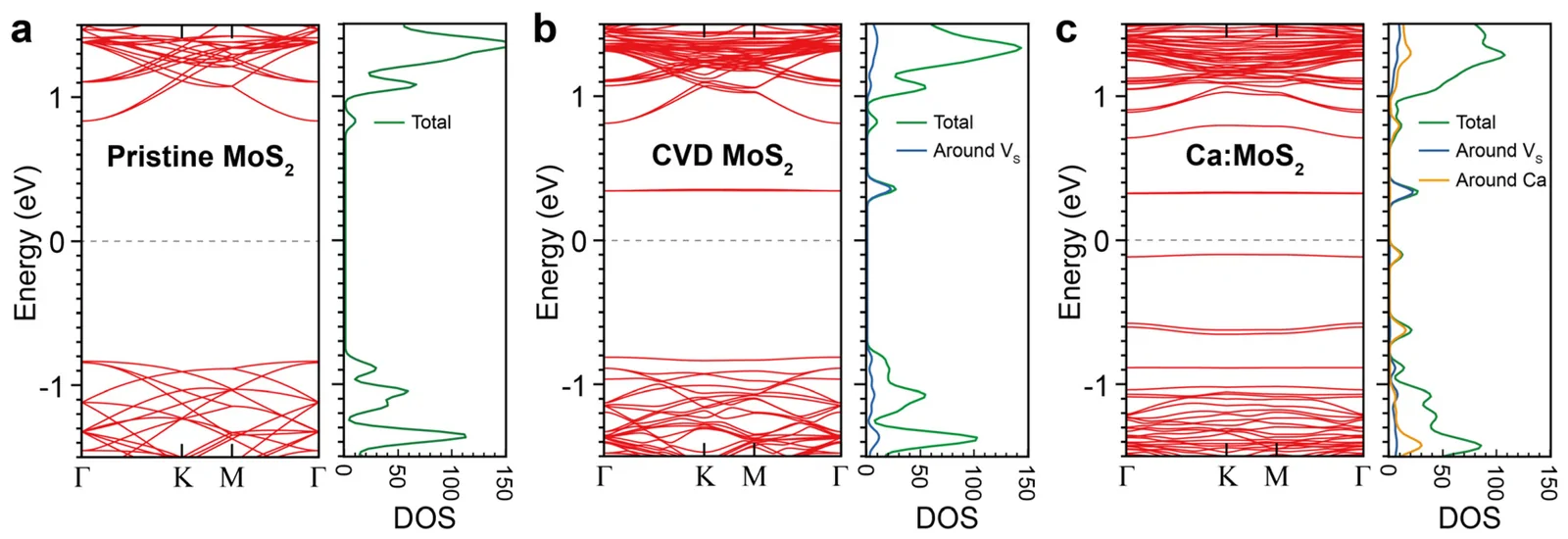
(**a**) Band structure and projected density of states (PDOS) of pristine monolayer MoS_2_ without S vacancies. (**b**) Band structure and projected density of states (DOS) of undoped monolayer MoS_2_ with one S vacancy. (**c**) Band structure and projected density of states (DOS) of Ca-doped monolayer MoS_2_ (Ca substituting one Mo atom) with one S vacancy.

To design an effective defect-engineering strategy, we first performed first-principles density functional theory (DFT) calculations to screen suitable dopants and predict their effects on the electronic structure of monolayer MoS_2_. The formation energy calculations reveal that Ca preferentially substitutes at the Mo site, indicating its experimental feasibility (Figure S1). Band structure calculations show that the pristine monolayer MoS2 exhibits a direct bandgap of 1.67 eV (Figure 1a). In contrast, the introduction of a sulfur (S) vacancy induces two deep donor levels below the conduction band minimum (CBM) (Figure 1b). The projected density of states (PDOS) calculations indicate that these two impurity levels are predominantly contributed by the atoms adjacent to the S vacancy site (see the right panel of Figure 1b), demonstrating that they are directly induced by the vacancy defect. These deep levels can act as nonradiative recombination centers and provide excess electrons, thereby severely quenching the PL emission. Remarkably, when a Ca atom substitutes a Mo atom in the presence of a sulfur vacancy, the Ca substitution introduces three acceptor levels that effectively counterbalance the donor levels from the sulfur vacancy (Figure 1c). This complementary defect-level engineering—where acceptor levels from Ca doping compensate donor levels from native S vacancies—suppresses nonradiative recombination and promotes the conversion of negative trions (X^−^) to neutral excitons (X^0^). These theoretical results establish a clear prediction: the synergistic interplay between Ca-induced complementary acceptor levels and native donor levels should suppress nonradiative recombination and promote exciton emission, leading to giant PL enhancement. This prediction is systematically verified through the experimental results presented below.

**Figure 2:**
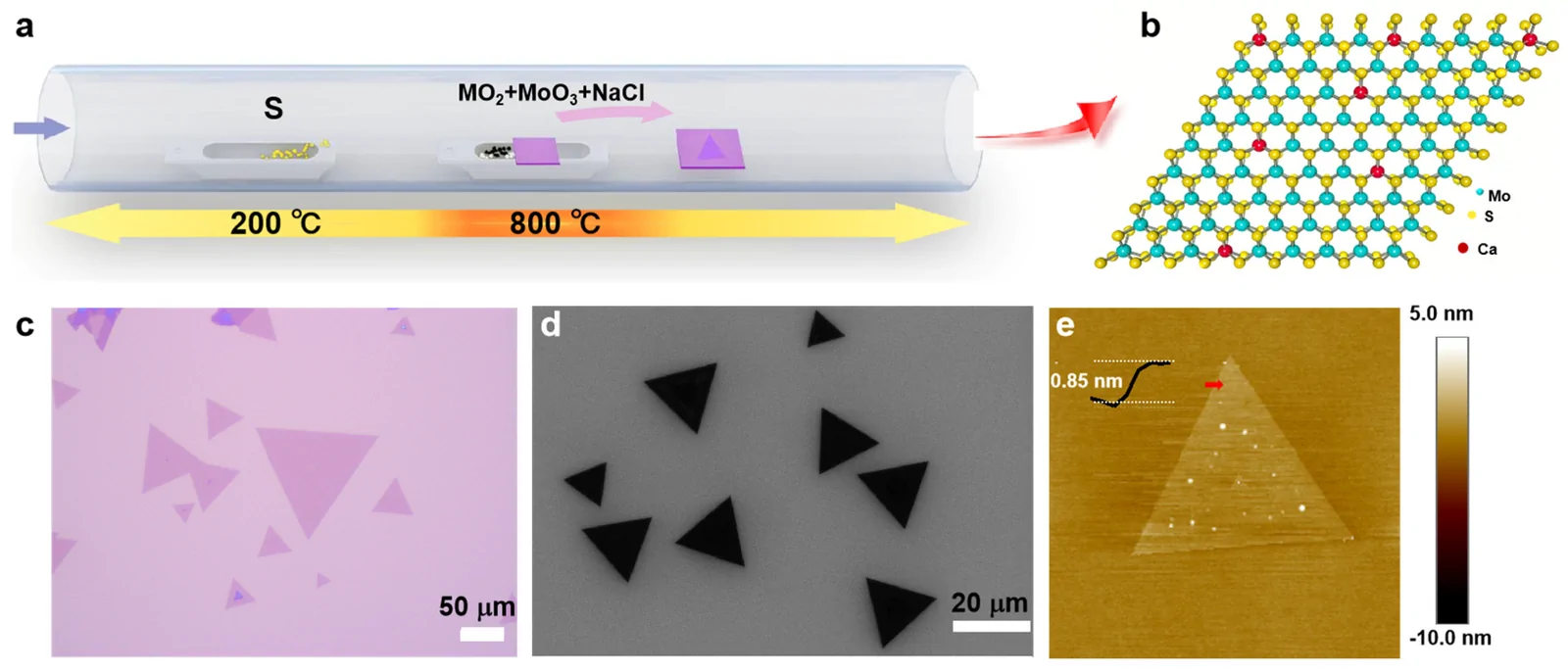
(**a**,**b**) Schematic diagram of the preparation process and atomic structure of the Ca:MoS_2_ monolayer. (**c**) Optical image of the Ca:MoS_2_. (**d**) SEM image of the Ca:MoS_2_ monolayer. (**e**) AFM image of the Ca:MoS_2_.

Guided by the DFT predictions, we synthesized Ca-doped monolayer MoS_2_ via a facile one-step CVD method. Figure 2a,b schematically illustrate the growth process and the corresponding atomic structure of Ca:MoS_2_, where Ca atoms substitute Mo sites in the MoS_2_ lattice. During the synthesis, a mixture of CaCl_2_, MoO_3_, and NaCl powders served as the precursor, with sulfur powder as the reactant (see Section 3 for details). The as-grown Ca:MoS_2_ samples exhibit typical triangular morphologies with clean surfaces and uniform thickness, as shown in the optical microscopy image (Figure 2c). Notably, the lateral size of the Ca:MoS_2_ flakes can reach up to sub-millimeter scale (~568 μm, Figure S2), which exceeds that of the vast majority of reported doped monolayer TMDCs and is favorable for practical device fabrication. The scanning electron microscopy (SEM) image in Figure 2d further confirms the large-area, continuous triangular morphology of the doped monolayer. Atomic force microscopy (AFM) characterization (Figure 2e) reveals a thickness of approximately 0.85 nm for the Ca:MoS_2_ flake, consistent with the typical thickness of monolayer MoS_2_, where the slightly larger value than the crystallographic thickness (~0.62 nm) is attributed to surface adsorbates and tip-convolution effects. These results demonstrate the successful synthesis of large-scale, high-quality Ca:MoS_2_ monolayers, providing a robust platform for subsequent optical and electronic investigations.

**Figure 3:**
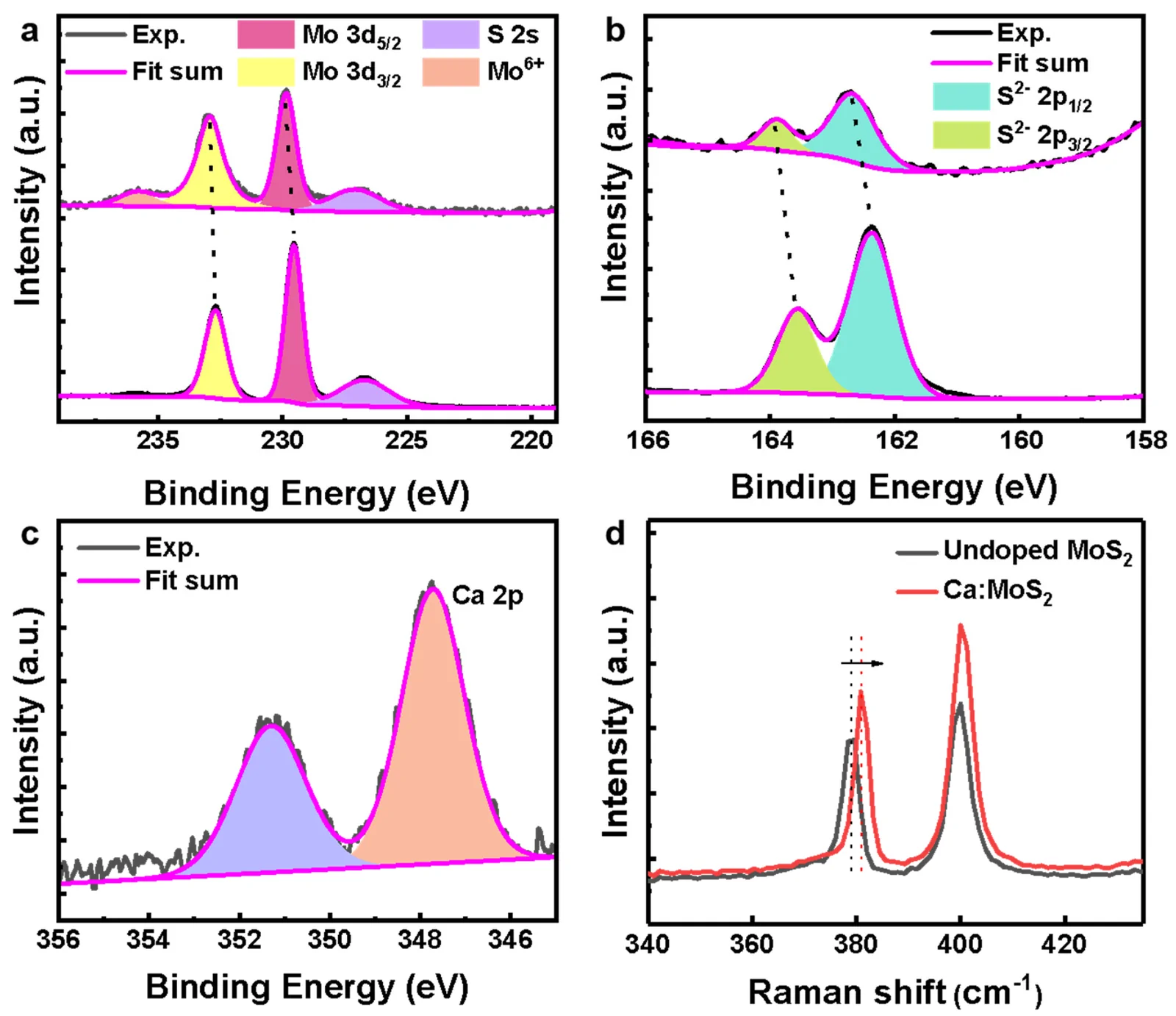
XPS scans of (**a**) Mo 3d, S 2s, (**b**) S 2p, and (**c**) Ca 2p core levels measured from undoped MoS_2_ and Ca:MoS_2_ samples. Raman spectra (**d**) of undoped MoS_2_ and Ca:MoS_2_ monolayers.

To further confirm the successful incorporation of Ca dopants and their influence on the electronic structure of MoS_2_, we performed X-ray photoelectron spectroscopy (XPS) and Raman spectroscopy measurements. Figure 3a,b show the high-resolution XPS spectra of Mo 3d, S 2s, and S 2p core levels for undoped MoS_2_ and Ca:MoS_2_ monolayers, respectively [35]. Compared with the undoped sample, both the S 2p and Mo 3d peaks in Ca:MoS_2_ shift to lower binding energies by 0.5 eV and 0.4 eV, respectively. This consistent negative shift indicates that the Fermi level of MoS_2_ moves toward the valence band upon Ca doping, demonstrating the expected p-type doping behavior of Ca substitution—consistent with Ca having fewer valence electrons than Mo. As expected, the Ca 2p core-level peaks (Ca 2p_3/2_ at ~347.59 eV and Ca 2p_1/2_ at ~351.14 eV) are exclusively detected in the Ca:MoS_2_ sample (Figure 3c), providing direct evidence for the successful incorporation of Ca atoms into the MoS_2_ lattice. XPS quantification reveals a Ca atomic percentage of approximately 2.8% in the Ca:MoS_2_ monolayer (Figure S3). This doping level was selected based on a systematic optimization of the MoO_3_:CaCl_2_ precursor ratio, where the 10:1 ratio (~2.8 at%) yields the most pronounced PL enhancement. Furthermore, Raman spectroscopy was employed to probe the vibrational properties of the doped samples. As shown in Figure 3d, the two characteristic Raman modes of monolayer MoS_2_—E^1^_2g_ (in-plane vibration) and A_1g_ (out-of-plane vibration)—are observed at ~379.6 and ~400 cm^−1^ for undoped MoS_2_. For Ca:MoS_2_, both peaks exhibit slight blue shifts to ~380.8 and ~400 cm^−1^, respectively. Such blue shifts are characteristic of p-type doping in TMDCs, further corroborating the p-type nature of Ca substitution. Collectively, the XPS and Raman results provide consistent and compelling evidence that Ca atoms have been successfully doped into the MoS_2_ lattice, inducing p-type doping behavior without degrading the monolayer structure.

**Figure 4:**
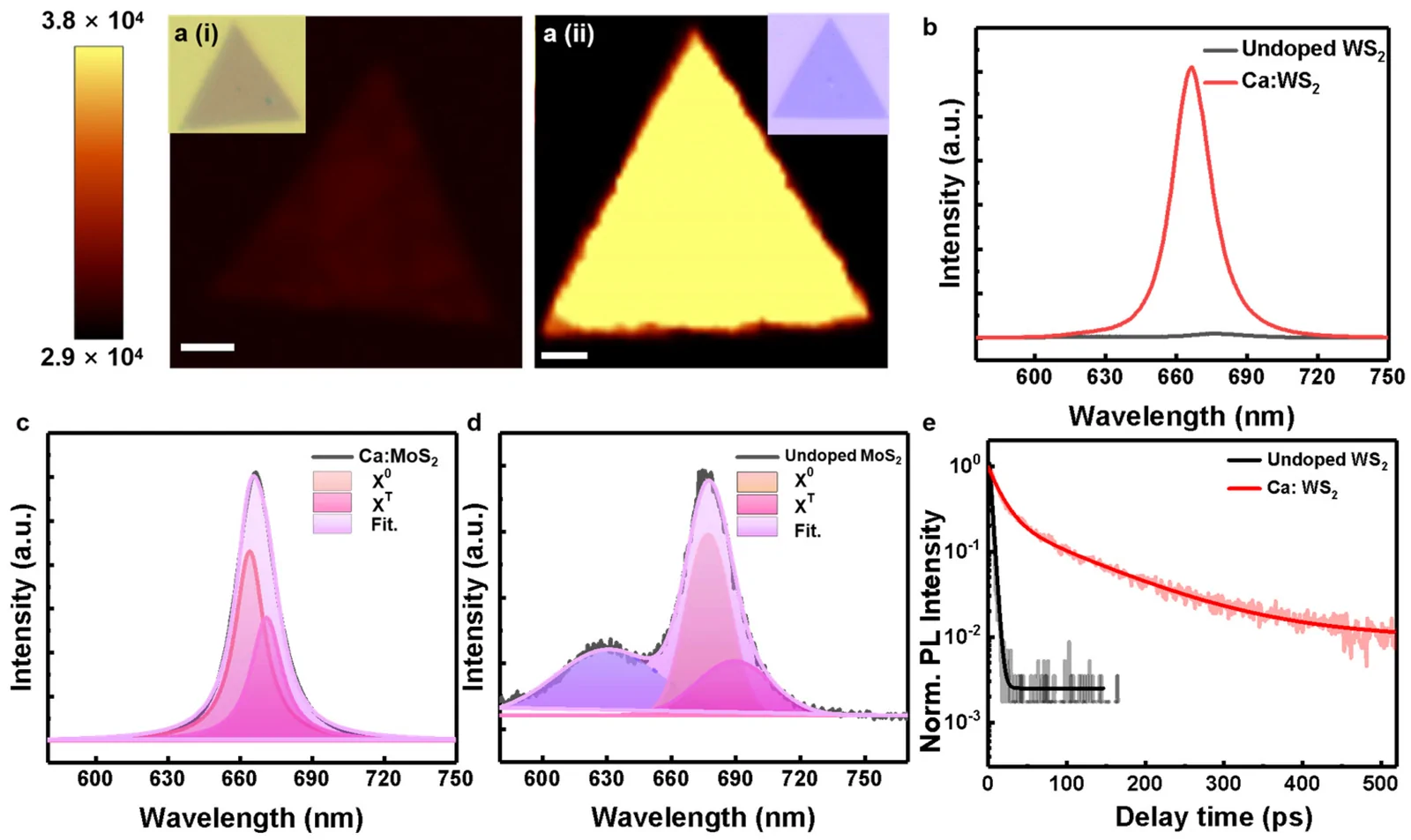
(**a**(**i**)) and Sr:MoS_2_ (**a**(**ii**)). The insets in (**a**(**i**,**ii**)) show the corresponding optical images. (**b**) PL spectra of undoped MoS_2_ and Ca:MoS_2_ monolayers. (**c**,**d**) Decomposed PL spectra of undoped MoS_2_ (**c**) and Ca:MoS_2_ (**d**), with the neutral exciton (X^0^) and trion (X^T^) components resolved by fitting. (**e**) Time-resolved PL (TRPL) decay spectra of undoped MoS_2_ and Ca:MoS_2_ monolayers.

To investigate the optical properties and verify the PL enhancement predicted by our DFT calculations, we performed spatially resolved PL mapping and spectroscopic measurements on undoped and Ca:MoS_2_ monolayers. Figure 4a(i,ii) show the integrated PL intensity maps of undoped MoS_2_ and Ca:MoS_2_, respectively (insets show the corresponding optical images). The Ca:MoS_2_ sample exhibits a dramatically brighter emission across the entire flake compared to the undoped counterpart. The enhancement is relatively uniform across the central region, with edge effects primarily attributed to the intrinsic edge states of MoS_2_. Figure 4b shows the PL spectra of undoped MoS_2_ and Ca:MoS_2_ monolayers. Remarkably, the Ca:MoS_2_ monolayer exhibits a giant PL enhancement of up to two orders of magnitude relative to the undoped sample (Figure S4), accompanied by a slight blue shift consistent with p-type doping behavior and a narrowed FWHM from ~38.9 nm to ~19.1 nm, indicating significantly improved radiative quality. To further verify the reproducibility of this enhancement, we performed additional PL measurements on six independently grown Ca:MoS_2_ samples. As shown in Figure S5, all doped samples exhibit significant PL enhancement compared to undoped MoS_2_, with enhancement factors ranging from approximately one to two orders of magnitude, confirming the effectiveness and reproducibility of our doping strategy. To elucidate the origin of this enhancement, we performed spectral deconvolution of the PL spectra. As shown in Figure 4c,d, the spectra can be well fitted with two components corresponding to neutral excitons (X^0^) and trions (X^T^). For undoped MoS_2_, the trion component dominates (~63.5%), reflecting the heavy n-type doping induced by sulfur vacancies. In striking contrast, the Ca:MoS_2_ sample exhibits a reversed trend: the neutral exciton fraction increases dramatically to ~59.4%, while the trion fraction decreases to ~40.6%. This pronounced trion-to-exciton conversion directly demonstrates that Ca doping effectively neutralizes excess electrons from sulfur vacancies. Moreover, ambient stability tests further reveal that the Ca:MoS_2_ monolayers exhibit significantly enhanced environmental stability compared to their undoped counterparts (Figure S6). Time-resolved PL (TRPL) measurements further corroborate this scenario (Figure 4e, Table S1). The Ca:MoS_2_ sample shows a significantly prolonged carrier lifetime (~38.0 ps) compared to undoped MoS_2_ (~3.1 ps), indicating effective suppression of nonradiative recombination pathways. Moreover, we found that this defect-engineering strategy is not limited to the Ca:MoS_2_ system; similar significant PL enhancement is also observed in other alkaline earth metal-doped WS_2_ (e.g., Mg:WS_2_) and in Ca-doped WS_2_ monolayers (Figure S7), demonstrating the generalizability of our complementary defect-level engineering approach across different TMDC host materials and dopant species. These optical results collectively validate our DFT-guided design: Ca doping introduces complementary acceptor levels that counterbalance donor levels from sulfur vacancies, suppressing nonradiative recombination, promoting exciton emission, and achieving the predicted giant PL enhancement.

## 3. Experimental Section

Materials Synthesis: The Ca-doped MoS_2_ monolayer was synthesized via a facile one-step CVD method at atmospheric pressure. A mixture of NaCl, MoO_3_ (20 mg), and CaCl_2_ (2 mg) was used as the precursor, with sulfur powder (99.9%, Alfa Aesar, Ward Hill, MA, USA) as the reactant. The addition of NaCl reduces the melting temperature of the reactants, promotes the generation of intermediate species, and consequently accelerates the reaction. The precursor powders were thoroughly mixed and placed in a porcelain boat at the center of the tube furnace. A SiO_2_/Si substrate was placed face-down above the mixture, and the sulfur source was positioned 13 cm upstream from the high-temperature zone. Prior to heating, the system was purged with Ar at 600 sccm for 18 min. The furnace was then heated to 800 °C within 30 min and held for 5 min, while the sulfur powder was heated to 200 °C. Ar at 120 sccm was used as the carrier gas throughout the growth. After growth, the furnace was naturally cooled to room temperature, yielding high-quality, large-scale Ca:MoS_2_ monolayers. Undoped MoS_2_ was synthesized under identical conditions except that CaCl_2_ was omitted.

Optical measurements: Raman and photoluminescence (PL) spectra of the Ca:MoS_2_ monolayers were collected using a confocal microscope (WITec, alpha-300, Ulm, Germany, part of Oxford Instruments Group) with a 532 nm laser at an excitation power of 75 μW. Time-resolved PL (TRPL) measurements were performed on the same confocal system equipped with a streak camera (Hamamatsu C10910, Hamamatsu, Japan), using a Ti–sapphire pulsed laser at 400 nm (pulse width: 80 fs, repetition rate: 80 MHz) as the excitation source.

First-principles Calculations: We performed first-principles density functional theory (DFT) calculations using the Vienna Ab initio Simulation Package (VASP). The exchange-correlation functional was treated within the generalized gradient approximation (GGA) as parameterized by Perdew, Burke, and Ernzerhof (PBE). The electron–ion interactions were described by the projector augmented-wave (PAW) method, and the plane-wave cutoff energy was set to 400 eV. A slab model with 6 × 6 × 1 supercells was employed to model the pristine, undoped, and Ca-doped MoS_2_ systems. All atomic positions were fully relaxed until the residual forces on each atom were less than 0.01 eV/Å and the total energy convergence reached 10^−6^ eV. The Brillouin zone was sampled using a Γ-centered 3 × 3 × 1 k-point mesh, and a vacuum layer of at least 20 Å was introduced to prevent spurious interactions between periodic images. The unfolding of band structures was performed using the KPROJ package (version: kproj-develop) based on the k-projection method. The formation energy (*E_form_*) is calculated using the equation *E_form_* = *E*(X_n_Y_m_) − *nμ_X_* − *mμ_Y_*, where *E*(X_n_Y_m_) represents the total energy of the X_n_Y_m_ compound, and *μ_X_* and *μ_Y_* denote the chemical potentials of the constituent atoms X and Y, respectively.

## 4. Conclusions

In summary, we have demonstrated a rational defect-engineering strategy for monolayer MoS_2_ through calcium doping, guided by first-principles DFT calculations and experimentally realized via a facile one-step CVD method. The Ca dopants introduce complementary acceptor levels that effectively counterbalance the native donor levels from sulfur vacancies, enabling controlled carrier dynamics with suppressed nonradiative recombination and efficient conversion of negative trions to neutral excitons. As a result, the Ca:MoS_2_ monolayers exhibit up to two orders of magnitude PL enhancement, significantly prolonged carrier lifetime, narrowed linewidth, and sub-millimeter lateral sizes (~568 μm). This strategy is also extendable to other dopants, demonstrating its generalizability as a versatile defect-engineering approach for 2D semiconductors. Our work not only provides a practical pathway for dramatically improving the optical quality of monolayer MoS_2_ but also establishes a fundamental framework—from theoretical prediction to experimental validation—for designing defect-engineered 2D materials with tailored optoelectronic functionalities, paving the way for high-performance integrated photonic and optoelectronic devices.

## Data Availability

The data that support the plots in this paper and other findings of this study are available from the corresponding authors upon reasonable request. Source data are provided with this paper.

## Supplementary Materials

The following supporting information can be downloaded at https://www.mdpi.com/article/10.3390/molecules31173073/s1, Figure S1: Formation energies of Ca: MoS_2_ monolayer; Figure S2: Large-scale optical microscopic image of the doped MoS_2_ monolayer; Figure S3: The PL and XPS spectrum of Ca:MoS_2_ monolayer; Figure S4: PL spectra of undoped MoS_2_ and Ca:MoS_2_ monolayers; Figure S5: PL spectra of undoped MoS_2_ and six independently grown Ca:MoS_2_ monolayers; Figure S6: Temporal stability test of samples under unprotected environmental conditions; Figure S7: Extension of universality: PL spectroscopy studies of Ca: WS_2_, Mg: WS_2_ monolayers; Table S1: PL lifetimes from TRPL dynamics of undoped MoS2 and Ca:MoS2 monolayers.
