# Supplementary material for: Rational Defect Engineering via Calcium Doping for High-Efficiency Monolayer MoS2 Emission
Source: Molecules. 2026 Sep 1;31(17):3073. doi: 10.3390/molecules31173073 (PMC13567196; doi:10.3390/molecules31173073)
Supplement: Supplementary file 1 [file molecules-31-03073-s001.zip › molecules-4467786-supplementary.pdf]

Supplementary Material

Rational defect engineering via calcium doping for high-efficiency monolayer MoS_2 emission

Ying Chen, Guoliang Yu, Yihua Hu, Xin Yang, Youlong Chen, Jingwen Zou, Haoqi Luo, Fangjie Li, Yushuang Zhang*, Qing Ye**

Dr. Y. Chen, Prof. Y.H. Hu, Dr. Y.L. Chen, J.W. Zou, Dr. H.Q. Luo, Dr. F.J. Li, Dr. Y.S. Zhang, Prof. Q. Ye
State Key Laboratory of Pulsed Power Laser Technology, Key Laboratory of Electronic Restriction of Anhui Province, National University of Defense Technology, Advanced Laser Technology Laboratory of Anhui Province, Hefei 230037, P. R. China.
Email: skl_hyh@163.com; yszhang@hnu.edu.cn, yeqing18@nudt.edu.cn.

Dr. G.L. Yu
School of Physics and Electronics, Hunan Normal University, Changsha, Hunan 410081, China.

Dr. X. Yang
State Key Laboratory of Opto-Electronic Information Acquisition and Protection Technology, Information Materials and Intelligent Sensing Laboratory of Anhui Province, Institutes of Physical Science and Information Technology, School of Physics, Anhui University, Hefei 230601, China

Section S1. Formation energies of Ca: MoS_2 monolayer.

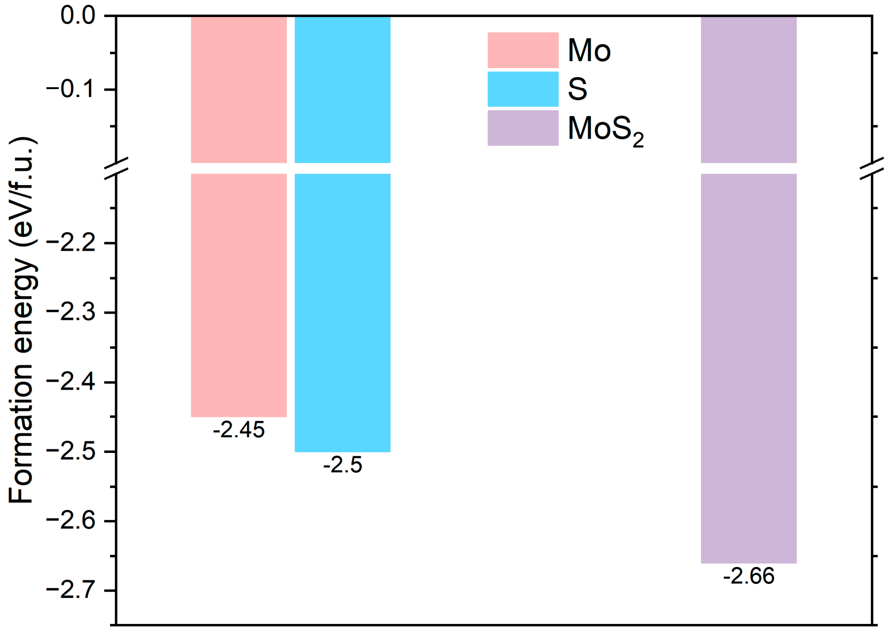

Figure S1. Formation energies of Ca atoms substituting for Mo and S atoms in the monolayer MoS_2 with an S vacancy, and the formation energy of the pristine monolayer MoS_2.

Section S2. Large-scale optical microscopic image of the doped MoS_2 monolayer.

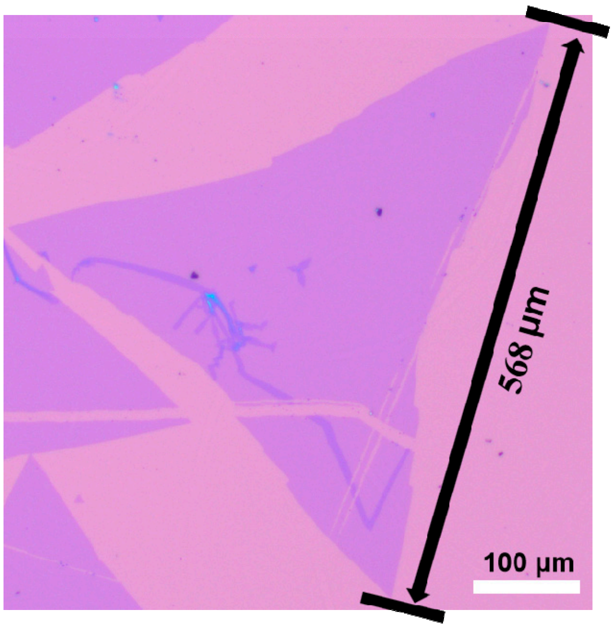

Figure S2. Optical microscopic image of the doped MoS_2 sample. Scar bar: 100 μm.

Section S3. The PL and XPS spectrum of Ca:MoS_2 monolayer.

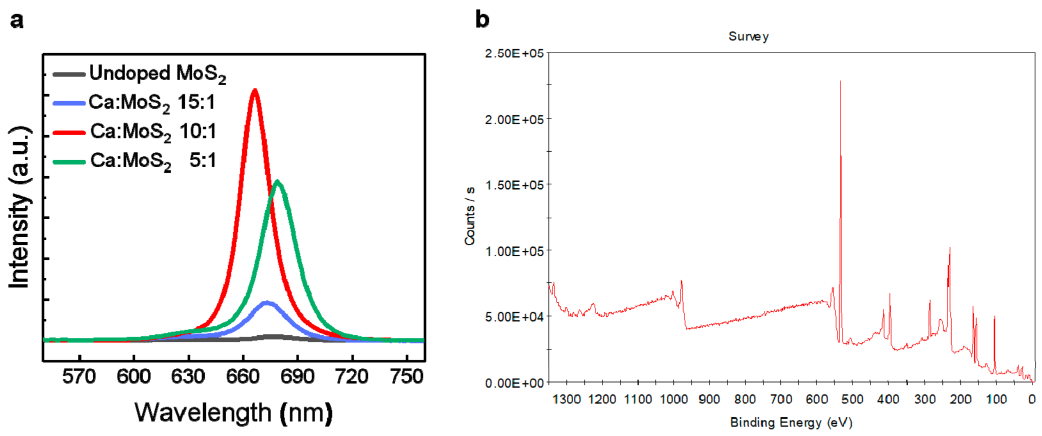

Figure S3. (a) PL spectra of Ca:MoS_2 monolayers prepared with different MoO_3:$CaCl_2$ precursor ratios (15:1, 10:1, and 5:1), compared with undoped MoS_2. (b) XPS spectrum of the Ca:MoS_2 sample prepared with the 10:1 precursor ratio, showing a Ca atomic percentage of approximately 2.8 at%.

Section S4. PL spectra of undoped MoS_2 and Ca:MoS_2 monolayers.

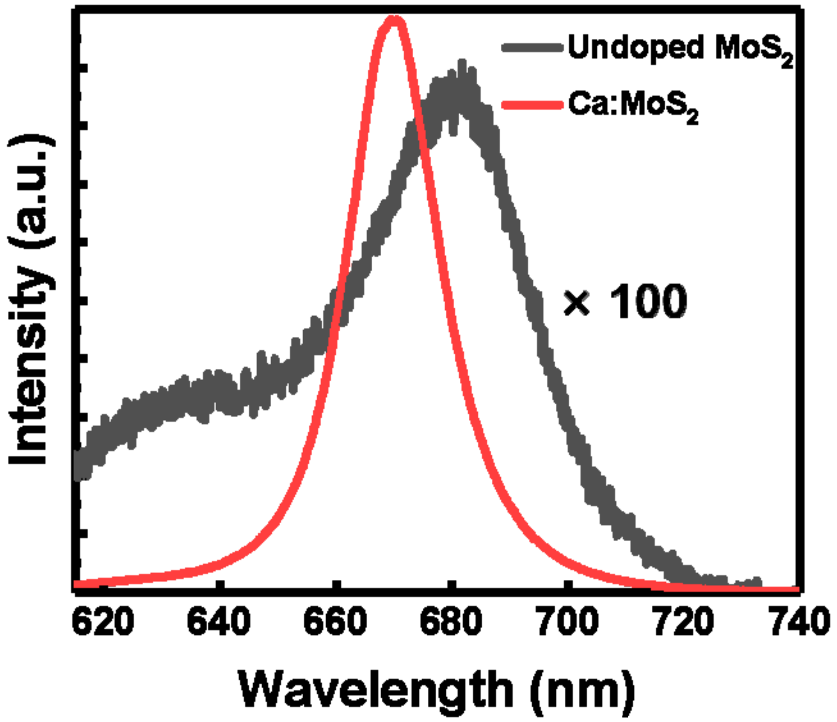

Figure S4. PL spectra of undoped MoS_2 and Ca:MoS_2 monolayers.

Section S5. PL spectra of undoped MoS_2 and six independently grown Ca:MoS_2 monolayers.

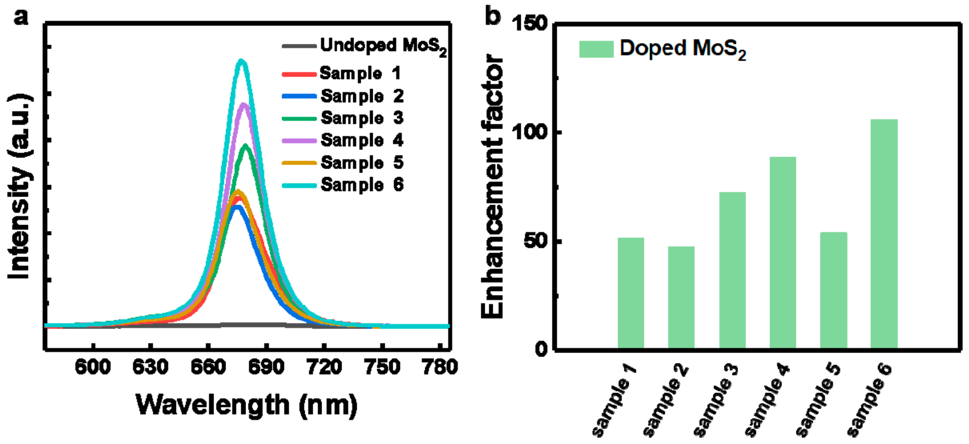

Figure S5. (a) PL spectra of undoped MoS_2 and six independently grown Ca:MoS_2 monolayers, showing consistent PL enhancement across different samples. (b) Corresponding PL enhancement factors of the six Ca:MoS_2 samples relative to undoped MoS_2.

Section S6. Temporal stability test of samples under unprotected environmental conditions

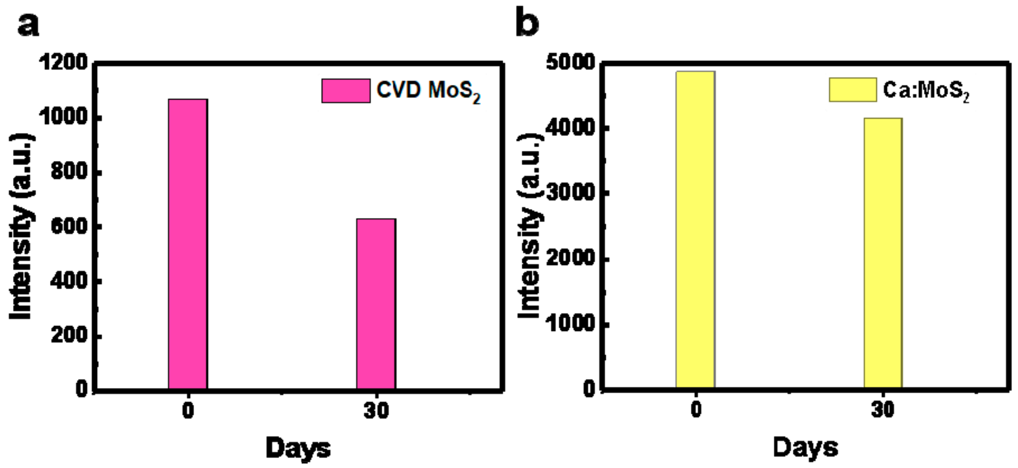

Figure S6. Time-dependent PL intensity of undoped MoS_2 (a) and Ca:MoS_2 (b) under ambient conditions over 30 days.

Section S7. Extension of universality: PL spectroscopy studies of Ca: WS_2, Mg: WS_2 monolayers.

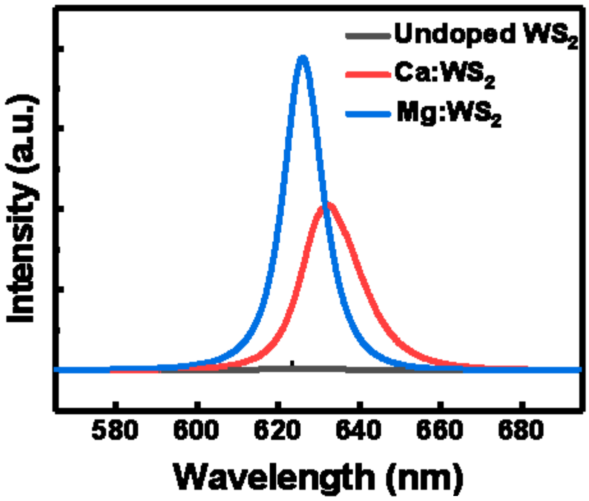

Figure S7. PL spectra of undoped WS_2, Ca:WS_2 and Mg:WS_2 monolayers.

Section S8. PL lifetimes from TRPL dynamics of undoped MoS_2 and Ca:MoS_2 monolayers

Specifically, the decay curves were fitted using:

Undoped MoS_2 (single-exponential):

$I(t) = A_0 \cdot \exp(-t/\tau_0) + y_0$

Ca:MoS_2 (biexponential):

$I(t) = A_1 \cdot \exp(-t/\tau_1) + A_2 \cdot \exp(-t/\tau_2) + y_0$

The average lifetime for the biexponential fit was calculated as:

$\tau = \tau_1 \cdot a_1(\%) + \tau_2 \cdot a_2(\%)$

Table S1. PL lifetime components and the averaged PL lifetimes of undoped MoS_2 and Ca:MoS_2 monolayers. τ_1 and τ_2 represent the resolved lifetime components. a_1 and a_2 are the corresponding proportions of these components. τ represents the averaged PL lifetime.

	τ_1 (ps)	a_1(%)	τ_2 (ps)	a_2 (%)	τ (ps)	R^2 (COD)
Undoped MoS_2	3.1	100			3.1	0.92
Ca:MoS_2	15.8	75.0	104.5	25.0	38.0	0.99
